# Current and previous spatial distributions of oilseed rape fields influence the abundance and the body size of a solitary wild bee, *Andrena cineraria*, in permanent grasslands

**DOI:** 10.1371/journal.pone.0197684

**Published:** 2018-05-22

**Authors:** Colin Van Reeth, Gaël Caro, Christian Bockstaller, Nadia Michel

**Affiliations:** 1 Laboratoire Agronomie Environnement (UMR 1121), Université de Lorraine, INRA, Vandœuvre-lès-Nancy, France; 2 Laboratoire Agronomie Environnement (UMR 1121), INRA, Université de Lorraine, Colmar, France; Philipps-Universitat Marburg Fachbereich Biologie, GERMANY

## Abstract

Wild bees are essential pollinators whose survival partly depends on the capacity of their environment to offer a sufficient amount of nectar and pollen. Semi-natural habitats and mass-flowering crops such as oilseed rape provide abundant floristic resources for bees. The aim of this study was to evaluate the influences of the spatial distribution of semi-natural habitats and oilseed rape fields on the abundance and the mean body size of a solitary bee in grasslands. We focused on a generalist mining bee, *Andrena cineraria*, that forages and reproduces during oilseed rape flowering. In 21 permanent grasslands of Eastern France, we captured 1 287 individuals (1 205 males and 82 females) and measured the body size of male individuals. The flower density in grasslands was quantified during bee captures (2016) and the landscape surrounding grasslands was characterized during two consecutive years (2015 and 2016). The influence of oilseed rape was tested through its distribution in the landscape during both the current year of bee sampling and the previous year. Bee abundance was positively influenced by the flower density in grasslands and by the area covered by oilseed rape around grasslands in the previous year. The mean body size of *A*. *cineraria* was explained by the interaction between flower density in the grassland and the distance to the nearest oilseed rape field in the current year: the flower density positively influenced the mean body size only in grasslands distant from oilseed rape. *A*. *cineraria* abundance and body size distribution were not affected by the area of semi-natural habitats in the landscape. The spatial distribution of oilseed rape fields (during both the current and the previous year) as well as the local density of grassland flowers drive both bee abundance and the mean value of an intraspecific trait (body size) in permanent grasslands. Space-time variations of bee abundance and mean body size in grasslands may have important ecological implications on plant pollination and on interspecific interactions between pollinators. Specifically, a competition between bee species for nesting sites might occur in oilseed rape rich landscapes, thus raising important conservation issues for bee species that do not benefit from oilseed rape resources.

## Introduction

Bees represent approximately 20 000 species worldwide that participate to the pollination of 87.5% of angiosperm species [1,2]. This monophyletic group is characterized by a large variety of morphological, phenological and behavioural traits. These functional traits not only differ from one species to another, but can vary at the intraspecific level [3,4]. For instance, foraging range is known to be highly variable at both the interspecific level [5,6] and at the intraspecific level [7]. Foraging range was related to body size in bees at the interspecific level [5,6], but may also vary with intraspecific body size variation as was shown for pollination efficiency [8,9]. Since bee individuals within species can have various foraging ranges, they differ in the way they respond to the resources located in the surrounding landscape [7].

The distribution of floristic and nesting resources in the landscape are major drivers of bee diversity and abundance in agro-ecosystems [10–12]. Semi-natural habitats (hereafter referred to as “SNH”) are essential habitats for bees providing both resources [1,13]. For instance, permanent grasslands with floristically dense or diverse vegetation are known to support abundant and diverse bee communities [14,15]. Oilseed rape (*Brassica napus*, hereafter referred to as “OSR”) is a mass-flowering crop that represents an alternative foraging habitat usable by some species emerging in early spring. Although the effects of habitat fragmentation on wild bee diversity have been intensively studied (e.g. [11]), the effects of OSR expansion in Europe (e.g. OSR surfaces in France quadrupled from 1970 to 2010 (FAOSTAT)) on wild bees and especially solitary bees are currently being assessed [16–18]. At the community level, Holzschuh et al. [16] found no influence of OSR area in the landscape on solitary bee abundance and diversity in SNH. Yet, it is known that some solitary generalist species are attracted by OSR massive nectar resources [17,19–21]. The effects of OSR on bees in SNH may be modulated by the quality of floral resources in SNH: OSR fields may be less attractive to bees when surrounding SNH contain a high flower density and diversity.

At the landscape scale, OSR expansion and SNH loss partly determine the spatial distribution of bees but their influence on intraspecific trait distribution of bees in agro-ecosystems is less documented. Warzecha et al. [22] found that the mean body size of two solitary bees *Andrena flavipes* (Panzer, 1799) and *Andrena haemorrhoa* (Fabricius, 1781) increased with SNH loss, suggesting a selection of larger individuals to reach remaining resourceful habitats. To our knowledge, OSR effects on intraspecific body size has not been studied yet. If we consider that all individuals in one species are attracted by OSR, we should expect that reaching a distant OSR field from the nest may be easier for larger individuals (i.e. with a large foraging range) than for smaller individuals. In addition to the effects of OSR in the current year, OSR fields in the previous year may also influence species distribution in the following year. Some species attracted by flowering OSR such as *Osmia bicornis* (Linnaeus, 1758) collect a larger amount of resources in OSR rich landscapes which in turn influences the production of offspring active in the following year [19,20]. This effect is known as the “productivity effect” [23].

In this context, the aim of our study was to assess the influence of both SNH area (current year) and OSR area (previous and current year) on a solitary bee in permanent grasslands. To achieve this, we considered both the abundance and the body size distribution of *Andrena cineraria* (Linnaeus, 1758), a large solitary bee species that forages both on SNH and OSR flowers. We hypothesized that (i) Grasslands with high flower density attract more individuals than flower-poor grasslands; (ii) SNH loss in the landscape decreases the abundance of *A*. *cineraria* in permanent grasslands; (iii) *A*. *cineraria* abundance in permanent grasslands is positively influenced by the surrounding area covered OSR in the previous year (i.e. “productivity effect” at the landscape scale); (iv) Proximity to flowering OSR fields positively affects bee abundance in grasslands (i.e. “attractiveness effect"); (v) Abundance loss with increasing distance to flowering OSR fields or decreasing area covered by SNH is driven by the loss of small individuals with lower flight capacity, so that mean body size increases in grasslands with a low accessibility.

## Materials and methods

### Study area and study sites

The study was carried out in 2016 in the “Parc Naturel Régional de Lorraine”, Lorraine, France (48°48’46” N, 6°43’14” E). This region covers a heterogeneous area of about 58 000 ha mainly composed of forests (33.7%), agricultural lands (annual crops (28.7%) and permanent grasslands (21.9%)). A total of 21 permanent grasslands were selected as study sites. Grasslands were chosen so that they were similar in size (mean ± standard deviation = 4.4 ± 1.2 ha), had extensive management with late mowing (June) and low nitrogen input (< 40 kg of nitrogen per year), and were highly heterogeneous regarding their surrounding landscape. The study sites represent a gradient in the area of SNH (4.4 to 58.4%) and OSR (2015: 0 to 19.6%; 2016: 0 to 18.0%) in a 900 m radius. The mean distance between study sites was 4 213 ± 1 730 m (min: 2 132 m; max: 8 214 m).

This study was carried out in private lands. Owners gave us permission to access their land.

### Study species: *Andrena cineraria*

*Andrena cineraria* (Linnaeus, 1758), known as the ashy mining bee, is a common species in Europe. Early individuals of the species emerge in late March and the peak activity occurs between April and May, during OSR flowering. At this time, females build nests in the soil and collect nectar and pollen for larval provisioning. After larvae pupate, they hibernate until the next spring. *A*. *cineraria* is known to be highly polylectic foraging on a wide range of plant family such as Apiaceae, Asteraceae, Brassicaceae, Ranunculaceae, Rosaceae, Salicaceae [24]. OSR flowers are known to be visited by both females and males of *A*. *cineraria* [17,25,26].

### Bee sampling and floristic characterization

During OSR flowering, we sampled bees during 14 days between mid-April and early-May 2016. We used pan traps (ProPac, Vechta, Germany) painted with UV bright (blue, white and yellow) colours (Sparvar Leuchtfarbe, Spray-Color GmbH, Merzenich, Germany) to maximize captures [27]. Traps were emptied three times in each grassland. Samplings were carried out during suitable conditions: sunny weather, no rain, and little wind (< 3 Beaufort). All captured *A*. *cineraria* were identified and counted. No specific authorization was required for this study that did not involve a protected species.

In each grassland, we quantified the mean flower density by counting the number of flower units in ten 1 m^2^ quadrats randomly placed around the pan traps, in the centre of the grassland. Mean flower density was highly correlated with the number of flowering plant species assessed in the same quadrats (Pearson correlation (r) = 0.84, *P* < 0.001).

### Bee functional characterization

To assess the foraging capacity of bee individuals, we measured the Inter Tegular Distance (ITD) on at least 10 male individuals in each grassland where possible. We chose to measure ITD only on male individuals because males were substantially more abundant than females, they are known to actively visit flowers [28] and they might have a greater body size heritability than females [29]. On average, ITD was measured for 41 individuals per site. Only three grasslands had less than 10 measures with 4, 4 and 6 individuals. These grasslands were kept in the analysis. For each grassland, we calculated the male mean body size of *A*. *cineraria* (ITD).

### Landscape characterisation

Gebhardt and Röhr [30] estimated the foraging distance of *A*. *cineraria* at 300 m. This value obtained by observation on host plants presumably underestimates the typical foraging distance, which according to Greenleaf et al. [6] equation is about 600 meters [31]. Moreover, the maximum foraging range (i.e. maximum feeder training distance) is likely to substantially exceed 600 m. Consequently, we assessed the landscape composition at three radii around each site (300, 600 and 900 m buffers) in 2015 and 2016. Hedgerows, woodlots and forests surfaces were extracted from BD TOPO® (IGN) whereas agricultural lands (crops, OSR, temporary grassland, orchard meadows, and permanent grasslands) were characterized yearly by field inspection. We calculated three landscape descriptors: (i) the SNH area in the landscape (hereafter referred to as “%SNH”), which included permanent grasslands, orchard meadows, hedgerows, woodlots and forest margins (10 meters wide); (ii) the OSR area in the landscape (hereafter referred to as “%OSR”). We focused on %OSR in 2015 (the year previous to the sampling) to test the “productivity effect” of OSR on bee abundance in the following year [23]; (iii) the distance between the centroid of each studied grassland (location of bee sampling) and the nearest OSR field edge (hereafter referred to as “distance to OSR”). We calculated this distance in 2016 (current year of bee sampling) to evaluate the “attractiveness effect” of OSR in relation to the foraging range of the species under consideration.

### Data analysis

The abundance of *A*. *cineraria* (males and females pooled together) and the male Inter Tegular Distance (ITD) were modelled separately as the response variables. Our modelling procedure was divided into three steps.

We first aimed to select the spatial scale that had the strongest influence on the response variables. Therefore, we correlated each response variable with %OSR in the previous year and %SNH at the three spatial scales (300, 600 and 900 m) as suggested in previous studies [19,32]. For each response variable, landscape variables were selected at the scale which yielded the highest spearman correlation (S1 Table).

During the second step, a general linear model with a negative binomial distribution was used to account for the overdispersed data of *A*. *cineraria* abundance. ITD was analysed with a linear model. Each of the two models contained: flower density, %OSR in the previous year (N-1), %SNH, and distance to OSR in the current year. We also added the interaction between flower density and distance to OSR to test whether proximity to OSR modulates the attractiveness of flower dense grasslands. Pearson correlation (r) between %OSR in the previous year and distance to OSR in the current year was relatively high whatever the spatial scale considered (r = -0.43, -0.49 and -0.50 respectively at 300m, 600m and 900m) but was below 0.7, the threshold value to test collinearity proposed by Dormann et al. [33]. All other correlations between predictors included in models gave |r| < 0.43. We tested the multi-collinearity between predictors in each model with Variance Inflation Factor (VIF; [34]) and found no variance inflation pattern since VIF < 1.82. Considering VIF = 3 as a threshold value [34], multi-collinearity was not an issue.

In the final step, full models were simplified by excluding one by one non-significant variables (P > 0.1 from F test or Likelihood Ratio test) in backward stepwise selection [16,35–37]. We visually controlled the homogeneity of the variance and the normal distribution of the residuals for each model. The goodness-of-fit of each model was checked with adjusted-R^2^ (R^2^_adj_) for linear models and with Nagelkerke’s pseudo-R^2^ for the general linear model [38].

Across all sites, we tested and found no spatial autocorrelation among sites with respect to bee abundance (Moran’s I = -0.091, *P* = 0.29) and ITD (Moran’s I = -0.058, *P* = 0.83) [39]. To illustrate the effects of the interaction between the flower density and distance to OSR, we split our data set in two groups according to the mean value of the distance to OSR (mean = 501 m). We thus formed two groups of grasslands: one with a distance to the nearest OSR field shorter than 501 meters (N = 11, mean ± SD = 205.8 ±135.5), and another with a distance larger than 501 meters (N = 10, mean ± SD = 826.6 ± 188.3).

## Results

We captured 1 287 *A*. *cineraria* individuals: 1 205 males and 82 females. The ITD for males ranged from 1.66 mm to 2.71 mm while the mean ITD per grassland ranged from 2.05 mm to 2.22 mm (2.12 ± 0.04 mm, N = 21 grasslands).

The goodness-of-fit of models yielded pseudo-R^2^ = 0.38 and R^2^_adj_ = 0.43 respectively for the abundance of *A*. *cineraria* and ITD models. These values indicated a reasonable model fit.

**Fig 1:**
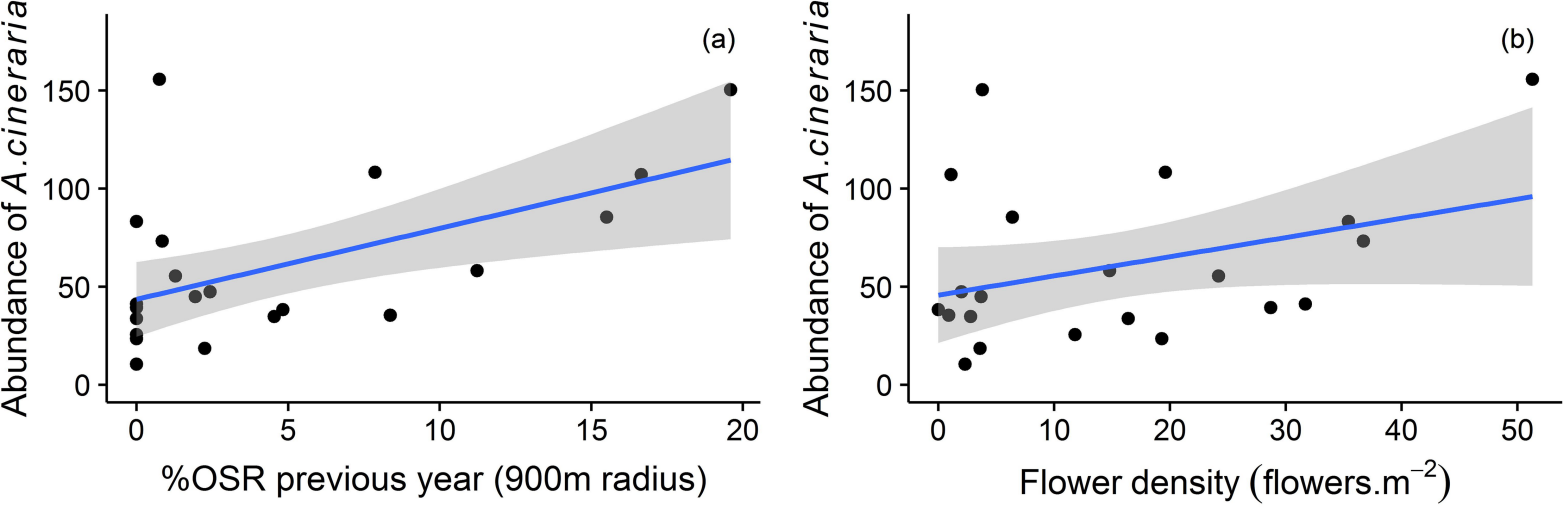
**Responses of the predicted abundance of *Andrena cineraria* in each grassland to (a) %OSR (900m radius) in the previous year (N-1) and (b) the flower density in grasslands**. Predictions returned by the abundance model are shown with the blue line. The grey band around the line represents the 95% confidence interval. (a) y = 43.611 + 3.620 * %OSR in the previous year (R^2^_adj_ = 0.261); (b) y = 45.762 + 0.979 * flower density (R^2^_adj_ = 0.078). Other statistical details are presented in Table 1. OSR = Oilseed rape.

The abundance of *A*. *cineraria* was positively influenced by the %OSR in the previous year and by the flower density in permanent grasslands (Table 1). The abundance of *A*. *cineraria* increased by 125% when the %OSR in the previous year increased from 0 to 15% (Fig 1A). Moreover, the abundance of *A*. *cineraria* increased by 107% when the flower density increased from 0 to 50 flowers m^-2^ (Fig 1B). %SNH only marginally affected abundance while the distance to OSR in the current year was not retained in the final model.

**Table 1:** Results of *A*. *cineraria* abundance and ITD models.

	df	Estimate	t or z value	*P*
***A*. *cineraria* abundance**				
%OSR (N-1)– 900m	1	1.07e-01	2.955	0.003
%SNH– 900m	1	2.49e-02	1.695	0.090
Flower density	1	4.75e-02	2.871	0.004
**ITD**				
Distance to OSR	1	-5.88e-05	-2.350	0.031
Flower density	1	-1.23e-03	-1.744	0.099
Interaction(Flower density: Distance to OSR)	1	4.71e-06	3.646	0.002

t-value are presented for linear models whereas z-value are presented for the general linear model. ITD: Inter Tegular Distance; Distance to OSR: Distance between the studied grassland and the nearest OSR field in the current year (N); %OSR (N-1): Oilseed rape area in the previous year (N-1); %SNH: Semi-natural habitats area in the current year.

**Fig 2:**
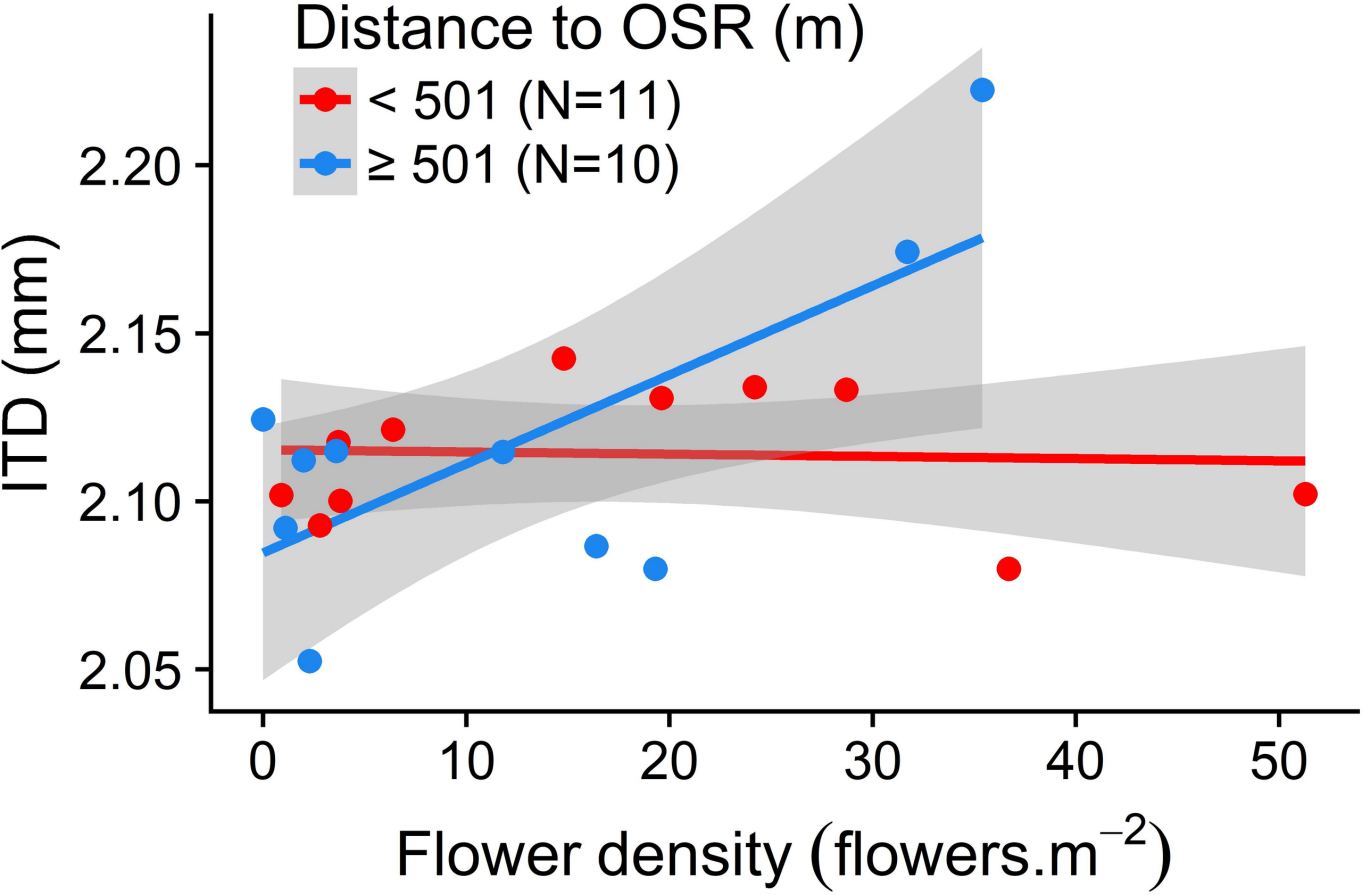
Interactive effect of the flower density and the distance to OSR on the mean ITD of *A*. *cineraria* in grasslands. To illustrate the interaction effect, data are presented for distance to OSR shorter than the mean = 501m (N = 11, mean ± SD = 205.8 ±135.5, red colour) and for distance to OSR larger than 501m (N = 10, mean ± SD = 826.6 ± 188.3, blue colour). The grey band around the line represents the 95% confidence interval. y = 2.115–6.562e-05 * flower density (Pearson correlation (r) = -0.05_,_ *P* = 0.875), y = 2.085 + 2.648e-03 * flower density (r = 0.71, *P* = 0.022). Other statistical details are presented in Table 1. ITD = Inter Tegular Distance; OSR = Oilseed rape.

ITD was significantly affected by the interaction between the flower density and the distance to OSR in the current year: flower density positively influenced ITD in grasslands distant to OSR fields (r = 0.71, *P* = 0.022) whereas flower density had no significant effect on ITD in grasslands close to OSR fields (r = -0.05, *P* = 0.875) (Table 1; Fig 2). %SNH and %OSR in the previous year did not influence ITD.

## Discussion

In this study, we demonstrated that the mean body size of a solitary bee species (*Andrena cineraria*) in grasslands was related to the grassland flower density and the proximity to oilseed rape. In addition, we highlighted that grassland flower density and the surrounding oilseed rape fields influenced the abundance of *A*. *cineraria*. More specifically, we showed that the area covered by oilseed rape in the previous year (i.e. productivity effect of oilseed rape at the landscape scale) was more important to explain *A*. *cineraria* abundance in grasslands than the proximity to oilseed rape in the current year (i.e. attractiveness effect).

Grasslands with high flower density attracted large *A*. *cineraria* individuals when grasslands were distant from the nearest oilseed rape field in the current year (Fig 2). Explanation for this observation is likely to rely on different flight capacity of small and large individuals. Only large individuals of this species can forage on both floristically dense grasslands and oilseed rape fields. When grasslands were close to an oilseed rape field, the flower density did not influence *A*. *cineraria* body size distribution probably because small and large individuals had the appropriate foraging ranges to reach oilseed rape fields and preferably foraged into these fields. Following this reasoning, the distance to the nearest oilseed rape field in the current year should have a negative effect on the abundance of *Andrena cineraria*. However, we did not observe such effect in our study. One possible explanation is that nearly all captured individuals were males. *Andrena* males not only forage but also spend a lot of time in mating and patrolling tasks [1,40]. Consequently, the influence of oilseed rape proximity may be more evident for females, which spend their time foraging for nectar and pollen to provision their offspring, than for males. The correlation between the distance to the nearest oilseed rape field and the female abundance seems to confirm this hypothesis (r (spearman) = -0.46; p = 0.04; N = 82). Unfortunately, because only 6% (N = 82) of our captured individuals were females, body size analyses were not possible. This local and landscape effects on the body size distribution may have important consequences on the pollination in grasslands because (i) large individuals visit more flowers per unit of time compared to small ones [41]; (ii) small and large individuals can visit different plant species [42]; (iii) large individuals carry larger pollen amounts than small individuals [8,43], but visit a significantly smaller plant spectrum [22]. Consequently, an optimal pollination service in grasslands may be reached not only when large individuals are present but also when a wide distribution of the body size occurs [8,42]. Pollinator-dependent crops such as oilseed rape also need large bee individuals: Jauker et al. [9] implemented a caged experiment and showed that oilseed rape yield was positively related to the body size distribution of *Osmia bicornis* individuals. Future studies are required to determine the optimal spatial organisation of habitats to maximize the pollination service in both semi-natural habitats and pollinator-dependent crops.

Long term effects of oilseed rape introduction in cropping systems on bee communities [23] and populations [18–20] have recently been taken into consideration. In this study, we focused on a solitary bee species foraging on oilseed rape flowers and whose reproduction occurs during oilseed rape flowering. We found that the abundance of *A*. *cineraria* was higher in grasslands surrounded by large areas covered by oilseed rape in the previous year than in grasslands surrounded by small areas of oilseed rape. This result is consistent with Holzschuh et al. [20] who showed that the number of brood cells constructed by *Osmia bicornis* during oilseed rape flowering was higher in grasslands adjacent to an oilseed rape field than in isolated grasslands (similar findings in Jauker et al. [19] and Dainese et al. [44]). In parallel, they found that the number of brood cells was positively correlated to the proportion of oilseed rape pollen in the larval food. These results indicate that the availability of oilseed rape resources may allow generalist species such as *O*. *bicornis* and *A*. *cineraria* to collect a larger quantity of resources and thus produce a larger number of larvae [19,20]. This effect at the population level might affect the bee community because the resulting high abundance in the following year could exacerbate the competition for resources with other species that are not attracted by oilseed rape [45]. Such competition might also occur after the period of oilseed rape flowering and affect wild plant pollination [46] when bee species benefitting from oilseed rape flower resources have a long foraging season or have several generations in one year (i.e. multivoltine species).

Even though body size is influenced by the resource availability at the larval stage [47–49], we did not find significant effect of oilseed rape area in the previous year on the mean body size of *A*. *cineraria*. This can be explained by the fact that body size not only depends on the quantity of resources provided during the larval stage but is likely to be also influenced by the resource quality [47,50]. In particular, Roulston and Cane [47] observed small bee individuals when fed with protein poor pollen during the larval stage and large bee individuals when fed with protein rich pollen. In the case of *Andrena cineraria*, oilseed rape pollen may not be optimal for larval development.

Habitat loss has been hypothesized to drive the intraspecific body size distribution of bees in agro-ecosystems. Warzecha et al. [22] found that the mean body size of two medium sized *Andrena* species (smaller than *A*. *cineraria*) increased with habitat loss, suggesting a selection for larger foraging ranges. However, *Andrena nigroaenea* (Kirby, 1802), a bee species whose size is similar to that of *A*. *cineraria*, was not influenced by habitat loss possibly because large species have the capacity to fly long distance between their nest and the remaining foraging sites [22]. Our results corroborate this assumption since no effect of habitat loss on the mean body size of the large species *A*. *cineraria* was found.

## Conclusions

This study contributes to the understanding of the ecological consequences of the expansion of oilseed rape areas across European landscapes on wild pollinators. Focusing on one solitary bee species, we showed that oilseed rape fields in the landscape influence both the abundance of *A*. *cineraria* in the following year (productivity effect of oilseed rape) and its body size distribution in the current year. Long-term consequences of an increasing abundance of bee species feeding on oilseed rape grasslands may have important ecological implications on wild plant pollination and on interspecific interactions between pollinators. Competition between bee species especially for nesting resources might occur in oilseed rape rich landscapes, thus raising important bee conservation issues.

## Supporting information

S1 Table Spearman correlations between response variables (Abundance of *Andrena cineraria* and ITD) and two landscape variables (%OSR in the previous year and %SNH) at three different spatial scales (300, 600 and 900m). (DOCX)

S2 Table Dataset. (DOCX)
