# Supplementary material for: Current and previous spatial distributions of oilseed rape fields influence the abundance and the body size of a solitary wild bee, Andrena cineraria, in permanent grasslands
Source: PLoS One. 2018 May 22;13(5):e0197684. doi: 10.1371/journal.pone.0197684 (PMC5963745; doi:10.1371/journal.pone.0197684)
Supplement: S1 Table — (DOCX) [file pone.0197684.s001.docx]

**S1 Table. Spearman correlations between response variables (Abundance of *Andrena cineraria* and ITD) and two landscape variables (%OSR in the previous year and %SNH) at three different spatial scales (300, 600 and 900m)**.

| Landscape variable | Radius (m) | Abundance of *A. cineraria* | | ITD | |
| --- | --- | --- | --- | --- | --- |
|  |  | r | *P* | r | *P* |
| %SNH | 300 | 0.12 | 0.61 | **0.16** | **0.49** |
| %SNH | 600 | -0.02 | 0.94 | -0.13 | 0.59 |
| %SNH | 900 | **0.12** | **0.60** | -0.02 | 0.94 |
| %OSR (N-1) | 300 | 0.36 | 0.20 | -0.09 | 0.71 |
| %OSR (N-1) | 600 | 0.29 | 0.11 | **0.12** | **0.59** |
| %OSR (N-1) | 900 | **0.40** | **0.07** | 0.02 | 0.94 |

For each response variable, the highest correlation coefficients (in bold) determine the best scale to consider for %SNH and %OSR (N-1). ITD: Inter Tegular Distance; %OSR (N-1): oilseed rape area in the previous year; %SNH: semi-natural habitats area.
