# Supplementary material for: Current and previous spatial distributions of oilseed rape fields influence the abundance and the body size of a solitary wild bee, Andrena cineraria, in permanent grasslands
Source: PLoS One. 2018 May 22;13(5):e0197684. doi: 10.1371/journal.pone.0197684 (PMC5963745; doi:10.1371/journal.pone.0197684)
Supplement: S2 Table — (DOCX) [file pone.0197684.s002.docx]

| **Grassland ID** | **Abundance A_cineraria** | **ITD** | **CV_ITD** | **Distance_OSR_2016** | **Floristic density** | **%SNH_300m** | **%SNH_600m** | **%SNH_900m** | **%OSR_300m_2015** | **%OSR_600m_2015** | **%OSR_900m_2015** |
| --- | --- | --- | --- | --- | --- | --- | --- | --- | --- | --- | --- |
| P106 | 22 | 2.13 | 4.95 | 478.8 | 24.2 | 35.65 | 16.15 | 27.29 | 0 | 3.98 | 1.28 |
| P108 | 26 | 2.09 | 5.51 | 503.4 | 1.1 | 28.68 | 34.89 | 31.93 | 0 | 4.67 | 16.64 |
| P109 | 37 | 2.12 | 5.71 | 95.3 | 6.4 | 11.7 | 9.86 | 17.61 | 24.84 | 19.7 | 15.5 |
| P116 | 26 | 2.09 | 6.66 | 135.5 | 2.8 | 27.99 | 37.71 | 35.46 | 0 | 2.25 | 4.54 |
| P125 | 6 | 2.22 | 8.71 | 1000 | 35.4 | 30.17 | 32.39 | 27.73 | 0 | 0 | 0 |
| P137 | 217 | 2.13 | 5.27 | 358.8 | 19.6 | 24.52 | 33.54 | 34.73 | 0 | 13.52 | 7.87 |
| P202 | 85 | 2.14 | 6.19 | 124.5 | 14.8 | 14.02 | 5.86 | 4.44 | 0 | 2.34 | 11.24 |
| P21 | 21 | 2.09 | 5.6 | 917.7 | 16.4 | 43.53 | 34.68 | 27.65 | 0 | 0 | 0 |
| P216 | 37 | 2.13 | 6.56 | 289.2 | 28.7 | 29.52 | 16.34 | 10.4 | 0 | 0 | 0 |
| P218 | 85 | 2.11 | 6.82 | 830 | 11.8 | 49.28 | 32.41 | 25.38 | 0 | 0 | 0 |
| P224 | 4 | 2.05 | 4.27 | 1000 | 2.3 | 6.4 | 8.28 | 7.57 | 0 | 0 | 0 |
| P228 | 68 | 2.17 | 8.03 | 823.1 | 31.7 | 9.94 | 7.28 | 6.55 | 0 | 0 | 0 |
| P230 | 3 | 2.08 | 7.25 | 1000 | 19.3 | 6.41 | 9.05 | 7.57 | 0 | 0 | 0 |
| P231 | 6 | 2.08 | 2.63 | 149.4 | 36.7 | 20.23 | 20.78 | 16.47 | 0 | 0 | 0.85 |
| P233 | 88 | 2.1 | 6.21 | 35 | 0.9 | 33.29 | 28.86 | 23.33 | 26.79 | 15.99 | 8.37 |
| P235 | 164 | 2.1 | 7.29 | 232.3 | 3.8 | 29.98 | 23 | 27.74 | 4.77 | 18.69 | 19.59 |
| P29 | 268 | 2.1 | 5.71 | 284.4 | 51.3 | 12.96 | 8.72 | 19.3 | 0 | 0 | 0.76 |
| P66 | 37 | 2.12 | 5.31 | 80 | 3.7 | 44.71 | 55.29 | 55.14 | 0 | 0 | 1.94 |
| P75 | 61 | 2.11 | 5.1 | 972 | 2 | 54.37 | 52.41 | 58.43 | 0 | 0 | 2.43 |
| P80 | 11 | 2.12 | 5.04 | 585.6 | 0 | 44.91 | 42.88 | 43.38 | 0 | 6.27 | 4.83 |
| P83 | 15 | 2.12 | 6.73 | 634.1 | 3.6 | 25.78 | 17.59 | 18.42 | 0 | 3.98 | 2.25 |

**S2 Table. Dataset.**

ITD: Inter Tegular Distance; CV_ITD: coefficient of variation of ITD; %OSR: oilseed rape area in the landscape; %SNH: semi-natural habitats area in the landscape.
